# Research on Nutrition, Dental Caries Status Using Novel Methods, and Related Factors to Preschool Children in Rural Areas of Vietnam

**DOI:** 10.1155/2022/7363163

**Published:** 2022-05-30

**Authors:** Do Hong Cuong, Vu Van Tam, Hoang Quy Tinh, Le Thanh Do, Nguyen Trong Nghia, Hoang Cong Anh

**Affiliations:** ^1^Hanoi Metropolitan University of Vietnam, 98 Duong Quang Ham, Quan Hoa, Cau Giay, Hanoi 100000, Vietnam; ^2^University of Science,Vietnam National University-Hanoi, 334 Nguyen Trai, Thanh Xuan, Hanoi 100000, Vietnam; ^3^Hanoi National University of Education, 136 Xuan Thuy, Dich Vong Hau, Cau Giay, Hanoi 100000, Vietnam; ^4^Duy Tan University, 254 Nguyen Van Linh, Thac Gian, Thanh Khe, Da Nang 550000, Vietnam; ^5^Ha Dong Genaral Hospital, 2 Be Van Dan, Quang Trung, Ha Dong, Hanoi 100000, Vietnam; ^6^115 Nghe An General Hospital, 40 Xo Viet Nghe Tinh, Vinh, Nghe An 460000-470000, Vietnam

## Abstract

The study aims to examine correlations between nutrition status with different factors and dental caries of preschool children in rural areas of Vietnam. A big data based on a total of 690 children (356 boys and 334 girls), aged 2–5 years, living in Van Xuan commune, were thoroughly analyzed. Oral examinations were performed by dentists with the assistance of nursery teachers and the research team. Caries was diagnosed using criteria established by the International Caries Detection and Assessment System (ICDAS). The examined children and their parents responded to questions pertaining to dental hygiene practices. The nutrition status of preschool children was determined by the World Health Organization (WHO) standards in 2006. There are factors which have effects on the malnutrition status of children in the research. The prevalence of dental caries also contributed importantly to assess children's development. In this study, the stunting groups have a higher ratio of caries compared to the others. Children's morphology and nutritional status are associated with dental caries among the preschool children in Van Xuan commune, Vinh Tuong district, Vinh Phuc province.

## 1. Introduction

Many studies on humans proved that early malnutrition affects the formation of tooth and also increase liability for dental caries [1, 2]. Dental caries is defined as the existence of one or more decayed surfaces in any tooth. The etiology of this chronic disease was found to be the accumulation of plaque in the roots of the teeth or on the enamel surface that causes continuous damage to the enamel and dentin [3]. Plaque contains cariogenic bacteria, such as *Streptococcus mutans*, *Lactobacilli*, and *Actinomycetes*. In-deep pathological studies have found that dietary consumption (such as higher consumption of free sugars), malnutrition, and host factors strongly influence dental caries, which eventually causes aches and restlessness and reduces growth hormones in children [4, 5].

Poor oral health can cause various disadvantages to children's nutrition, growth, and development. Thus, it is necessary to identify the risk factors and take precautions for childhood caries. Public Health England [6] showed the correlation between obesity and dental caries, and Oliveira et al. [4] justified that dental caries was associated with social factors and nutritional status. Dung and Tuan [7] showed 81.4% of children with dental caries in 7775 children were 4–8 years. Gerdin et al. [8] and Hooley et al. [2] indicated that the dental caries status was in direct proportion to weight. In another work, Shen et al. [9] showed that dental caries had terrible effects on the development of children's height and nutrition status. Children's malnutrition often has many serious consequences, both physically and mentally. Children's stunting and being underweight is one of the fastest indicators reflecting their health and development status.

According to the WHO standards, there are four levels of both stunting and underweight malnutrition: low, average, high, and very high levels [10]. The dataset of malnutrition, dental caries status, and correspondence analysis was performed using SPSS 11.5 software.

Preschool children in Vietnam suffer from a “Double Burden” of malnutrition, in which the percentages of stunting, underweight, and wasted are still high (25.9%, 15.3%, and 7.9%, respectively). Meanwhile, the rate of obesity is increasing [7, 11]. This situation is popular in many places in developing countries, especially areas where economic development is starting, for example, Van Xuan commune, Vinh Tuong district, Vinh Phuc province. Therefore, the aim of the present study is to examine the relationships between dental caries and malnutrition status (other factors) in preschool children, aged 2–5 years, in Van Xuan commune, Vinh Tuong district, Vinh Phuc province.

## 2. Methods

A total of 690 preschool children aged 2–5 years, 356 boys (51.6%) and 334 girls (48.4%), and their parents living in Van Xuan commune, Vinh Tuong district, Vinh Phuc province, Vietnam, were examined. The children and their parents were recruited at three nursery schools. Babysitters (teachers) worked as partners of research, explained to the parents and children about the purpose and process of this research, and asked them if they agreed to participate. The teachers also accompanied the children during examinations. Before participating in the study, parents provided written consent and child assent for participation in the study.

None of the children were found to have an unusual indicator, so all collected data were used to analyze in this research. The indicators, such as date of birth, date of examination, sex, weight, and height of children, were inputted to the WHO AnthroPlus software which output the age of children and some nutritional indices to help assess the nutritional status of the children. Based on all output data by the WHO AnthroPlus software, SPSS 11.5 software was used to statistically evaluate the relationships between malnutrition and dental caries of the children in the study. Furthermore, some socioeconomic factors also were added to the SPSS 11.5 software to analyze the effect on nutrition and caries in children. Pearson correlation (*r*) was used to find the relation between the nutrition status and dental caries. In it, the value represents the strength of this relationship according to the following criteria [12]:

  |r| < 0.1: very weak relationship
  |r| < 0.3: weak relationship
  |r| < 0.5: moderate relationship
  |r| ≥ 0.5: strong relationship

### 2.1. Data Collection

Using prepackaged sterilized oral examination kits, a trained dentist examined children for dental caries and periodontal conditions. These examinations were performed in accordance with the ICDAS standards. In addition, the parents of children were administered a questionnaire that elicited demographic characteristics and information about oral hygiene practices, the amount and timing of dietary consumption, frequency of dental appointments, and parental evaluation of the child's teeth. The dental caries was determined based on the ICDAS [13].

Anthropometric indicators including weight and height for each age were collected. The body mass index (BMI) of each individual was calculated, while the nutritional status was determined according to the WHO standards in 2006.

## 3. Results and Discussion

Data derived from questionnaires and dental examinations were screened for abnormalities in any basic health indicators of any children.

Distributions of age and sex for the preschool children are given in Table 1. Of 690 children, 51.6% are male and 48.4% are female. The number of 2-year-old children of both two sexes is the lowest in the study, and 92.7% of the research is performed on 3–5 years old.

**Figure 1:**
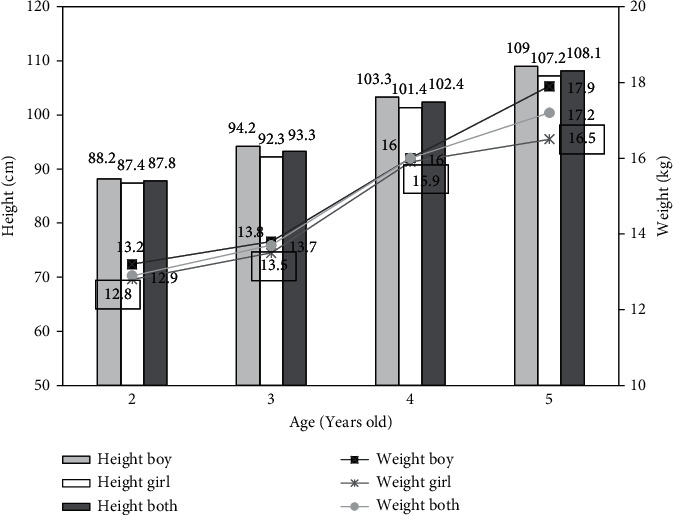
Average height (bars) and weight (lines) by sex and age of preschool children. The data for both sexes are presented with standard deviations.

Height and weight by age and sex of the preschool children are shown in Figure 1. The means of height and weight of boys were significantly higher than those of girls. These results suggest that normal development compared to other children in Vietnam is dominant. The averages of height and weight of children in this study were similar to the WHO standards for children of the same age and sex. The one increased fastest at the age of 3-4 years (height increased by 9.1 cm and weight increased by 2.3 kg).

### 3.1. Nutrition Status

The distribution of stunting malnutrition status in preschool children is given in Table 2. According to the WHO standards, the overall proportion of stunting of children in our study was at a low level. Table 2 provides that the rate of stunting malnutrition of children aged 2 and 3 years was at average levels of 28.0% and 28.8%, respectively. On the other hand, the properties of groups of 4 and 5 years old were low levels of 11.8% and 12.3%, respectively (Table 2).

The overall low weight of children in our study was found to be underweight, in which the proportion of underweight children in the group aged 2–4 years was at a low category (4.0–8.7%) and the rate of underweight children in the group of 5 years old was at the normal class with a lightweight ratio of 14.0% (Table 2).

Based on BMI-for-age, the proportion of wasted and obese children was found in low category. The average frequency of children residues of the 5 years old group had the highest standard of malnutrition with a wasted ratio of 6.1% and the lowest was the 3 years old group with an incidence rate of 1.9% (Table 2). The overweight rate of children at 2 years old was the highest (20%), but none of them were obese. Overweight ratios in other groups were 1.9%, 2.9%, and 7.9% for the groups of 3, 4, and 5 years old, respectively. There were ten children (4.8%) who were determined to be obese, and they were only in the 3 and 4 years old groups.

### 3.2. Some Factors Related to Nutrition Status

Based on answers to the questionnaires, some factors are related to the nutrition status of children given in Table 3, including the job of parents, educational level of parents, the number of children in a family, using water resources, and regularly eating sugar sweet.

Job is a factor related to families' economy, so it indirectly affects the nutrition status of children. For children whose parents were farmers, the risk of them being underweight and stunting was 1.6 and 1.2 times higher than those whose parents worked as government officers and business in this study, respectively. In addition, the education level of parents also was a trouble with the nutrition status of their children. Parents with high educational levels often have a better understanding and knowledge regarding taking care of nutrition for their children. In our research, the percentage of underweight children was 2.3 times higher than the children whose parents have an education level below the secondary school. Furthermore, families that have many children often do not take good care of kids' nutrition, especially those in poor areas. Table 3 provides that the underweight kids who live in families which have more than three children had a risk of the one and were 1.5 times higher than the families that have less than two children. The water source is also a serious problem with the children's physical health. 7.2% of the families were using natural water sources in their daily lives. For children of these families, the risk of being underweight was 1.45 times higher than that of the others. Related to the overweight-obesity status in this study, Table 3 also provides that 44.6% of children regularly eat sugar sweets. These children have a risk of overweight/obesity that was 1.45 times higher than those who do not or rarely eat sugar sweets.

### 3.3. Nutrition Status and Dental Caries

In our study, the overall rate of dental caries among preschool children was 71.3%, and it varied within other groups. Only 40% of 2 years old children had dental caries, which was much lower than the older ones.

The percentage of dental caries of children in the age groups of 3, 4, and 5 years old was 71.2%, 77.5%, and 72.8%, respectively (Table 4). The rate of dental caries in children aged 2 years was low, and the frequency of dental caries in children aged 3–5 was found at a moderate level [14]. The rates of dental caries in 4 categories of stunting, underweight, wasted, and overweight/obesity was 63.5%, 61.3%, 58.3%, and 58.3%, respectively (Table 4). The children with malnutrition have a higher ratio of dental caries in this study (statistically significant is the difference in the stunting group, *p* < 0.05).

### 3.4. Parent's Care on the Dental Issue

Based on further investigation of dental issues of children, we found that 662 (95.9%) children brushed their teeth at least once per day (Table 5). However, 132 parents (19.1%) thought their children did not have bad teeth, but in fact, their children had dental problems. There are 440 (63.8%) recruited children who had at least one bad tooth or more, with only 20 among them with the worst dental status [13]. Interestingly, only 234 parents (33.9%) reported that their children had a dental problem. Additionally, 114 children had never been checked for their dental status (Table 5).

Correlations between dental caries and the nutrition status of the children in this research were assessed by using the SPSS 11.5 software. The result is given in Table 6.

Table 6 provides that dental caries and weight-for-age of the children do not correlate (*p* > 0.05). Meanwhile, both height-for-age and BMI-for-age have a weak correlation with dental caries in this study (|*r*| < 0.3, *p* < 0.05). Our result is similar to the result of one of the research studies performed by Public Health England [6], which shows weak to average correlation between the increasing proportion of obesity and dental caries in children.

The sex ratio of children in our study was close to the human natural birth ratio and differed national birth ratio in Vietnam, in which 108 boys to 100 girls in the year 2019 was higher compared with previous years. Babies normally stay at home with their grandparents or babysitters up to 2 years old or even later. However, they have to spend at least one year to learn characters and numbers before primary school. This explains the high percentage of children at 4 or 5 years old.

The rate of overweight and obesity in children in our study (6.5%) was higher than that reported by the local authority of the Vinh Phuc province (4.7%), although these values were lower than nationwide (7%) values, reflecting the dual dietary burden of a developing country. The stunting condition was correlative with the higher development of dental caries (63.5%), which is similar to another study [15, 16]. Malnutrition could cause dental caries through the impact of tooth enamel formation and children's chewing ability [11]. The direct effect of dental caries, pain, and gingivitis is related to the child's ability to eat, and it leads to a poor diet, which contributes to slow weight gain and height increase [1, 17].

Overall, dental caries among children in our study was 71.3%, which ranked as a moderate level according to the WHO classification. A bad tooth at level 2 or 3 (in a 3-level system) can be easily recognized by any parent. But a high percentage of parents are unaware of their children's status, and the children never even had a dental check, showing that parent's care is the most important factor to maintain a good dental status for children in the rural areas of Vietnam, raising the need of social education for dental care in children.

## 4. Conclusions

The research used the WHO standards in 2006 to assess nutrition status and ICDAS to analyze caries. SPSS 11.5 software was used to analyze datasets and found a relationship between malnutrition status and dental caries and some factors of preschool children. The result of this study showed that the stunting, underweight, and wasted status of preschool children were, respectively, 18.3%, 9%, and 3.5% (low categories). The mean of the dental caries rate of the children was 71.3% (average categories). The factors related to the malnutrition status include the job of parents, educational level of parents, number of children in a family, using water resources, and eating sugar sweets. The stunting groups have a higher ratio of dental caries compared to the other groups. Parent's care is the most important factor to maintain a good dental status for children in the rural area of Vietnam; therefore, social education for dental care in children is a need. We suggest that there are need for important measures to reduce dental caries in Vietnamese preschool children in Van Xuan commune and rural communities with similar characteristics.

## Figures and Tables

**Table 1:** The percentage of the participated children by age and sex.

Age	Boy		Girl	
	*n*	%	*n*	%
2	22	3.2	28	4.1
3	112	16.2	96	13.9
4	110	15.9	94	13.6
5	112	16.2	116	16.8

Natural numbers (*n*) indicate the number of children in groups; the decimals indicate the percentages of the groups.

**Table 2:** Nutritional status of children.

Nutritional status of children based on height-for-age										
	Stunting		Normal							
Age	*n*	%	*n*	%						

2	14	28.0	36	72.0						
3	60	28.8	148	71.2						
4	24	11.8	180	88.2						
5	28	12.3	200	87.7						

Nutritional status of children based on weight-for-age										
	Underweight		Normal							
Age	*n*	%	*n*	%						

2	2	4.0	48	96.0						
3	18	8.7	190	91.3						
4	10	4.9	194	95.1						
5	32	14.0	196	86.0						

Nutritional status of children based on BMI-for-age										
	Wasted		Normal		Overweight risk		Overweight		Obesity	
Age	*n*	%	*n*	%	*n*	%	*n*	%	*n*	%

2	2	4.0	22	44.0	16	32.0	10	20.0	0	0.0
3	4	1.9	168	80.8	24	11.5	4	1.9	8	3.8
4	4	2.0	172	84.3	20	9.8	6	2.9	2	1.0
5	14	6.1	196	86.0	0	0.0	18	7.9	0	0.0

Natural numbers (*n*) indicate the number of children in groups; the decimals indicate the percentages of the groups.

**Table 3:** Some factors related to nutrition status.

Job of parents	Nutrition status				
	Underweight	Normal	Stunting		Normal
Farmer	50	456	96		410
Gov. officer, business	12	172	30		154
OR (95% CI)	1.6 (0.82–3.02)		1.2 (0.8–1.9)		

Educational level of parents					
	Underweight	Normal			
Below secondary school	8	38			
Above secondary school	54	590			
OR (95% CI)	2.3 (1.02–5.18)				

The number of children in a family					
	Underweight	Normal			
More than three	22	170			
One or two	40	458			
OR (95% CI)	1.5 (0.9–2.6)				

Using water resource					
	Underweight	Normal			
Natural	6	44			
Treated	56	584			
OR (95% CI)	1.45 (0.6–3.5)				

Regularly eating sugar sweet.					
	Overweight/obesity	Normal			
Regularly	24	484			
Do not or rarely	6	176			
OR (95% CI)	1.45 (0.6–3.6)				

**Table 4:** Dental caries of children in the study.

Dental caries by age of children in the study					
	Dental caries		Normal		
Age	*n*	%	*n*	%	

2	20	40.0	30	60.0	
3	148	71.2	60	28.8	
4	158	77.5	46	22.5	
5	166	72.8	62	27.2	
Total	492	71.3	198	28.7	

The dental caries status of malnutrition children in the study					
	Dental caries		Normal		
Nutritional status	*n*	%	*n*	%	*P*

Stunting	80	63.5	46	36.5	<0.05
Underweight	38	61.3	24	38.7	>0.05
Wasted	14	58.3	10	41.7	>0.05
Overweight/obesity	28	58.3	20	41.7	>0.05

Natural numbers (*n*) indicate the number of children in the groups; the decimals indicate the percentages of the groups.

**Table 5:** Parent's care about the dental issue of children.

Brushing teeth of children with or without help from parents per day			
1 time	2 times	More than 2 times	None
224	390	48	28

Checking the dental status of kids per year (self-evaluation and by the dentist)			
1 time	2 times	More than 2	None
210	238	128	114

Do the children have bad teeth?			
Yes	No	No answer	Not sure
234	432	22	2

**Table 6:** Correlation between dental caries and malnutrition status.

		Weight-for-age	Height-for-age	BMI-for-age
Dental caries status	Pearson correlation (*r*)	−0.070	−0.08	0.115
	*P*	0.068	0.032	0.002
	*N*	690	690	690

## Data Availability

The data used to support this study are included within the article.

## References

[1] Dye B. A., Thornton-Evans G., Li X., Iafolla T. J. (2015). Dental caries and sealant prevalence in children and adolescents in the United States, 2011–2012. *NCHS Data Brief*.

[2] Hooley M., Skouteris H., Millar L. (2012). The relationship between childhood weight, dental caries and eating practices in children aged 4–8 years in Australia, 2004–2008. *Pediatric Obesity*.

[3] Mishu M. P., Hobdell M., Khan M. H., Hubbard R. M., Sabbah W. (2013). Relationship between untreated dental caries and weight and height of 6-to 12-year-old primary school children in Bangladesh. *International Journal of Dentistry*.

[4] Oliveira L. B., Sheiham A., Bönecker M. (2008). Exploring the association of dental caries with social factors and nutritional status in Brazilian preschool children. *European Journal of Oral Sciences*.

[5] Psoter W. J., Reid B. C., Katz R. V. (2005). Malnutrition and dental caries: a review of the literature. *Caries Research*.

[6] Public Health England (2015). *The Relationship between Dental Caries and Obesity in Children: An Evidence Summary*.

[7] Dung T. M., Tuan V. M. (2011). Current situation of dental caries and some related factors in children aged 4-8 years old in 5 provinces and cities of Vietnam in 2010. *Journalism Practice Medicine*.

[8] Gerdin E. W., Angbratt M., Aronsson K., Eriksson E., Johansson I. (2008). Dental caries and body mass index by socio‐economic status in Swedish children. *Community Dentistry and Oral Epidemiology*.

[9] Shen A., Bernabé E., Sabbah W. (2019). The bidirectional relationship between weight, height and dental caries among preschool children in China. *PLoS One*.

[10] De Onis M., Blossner M., World Health Organization (1997). *WHO Global Database on Child Growth and Malnutrition*.

[11] Khanh L. N., Ivey S. L., Sokal-Gutierrez K. (2015). Early childhood caries, mouth pain, and nutritional threats in Vietnam. *American Journal of Public Health*.

[12] Field A. (2009). *Discovering Statistics Using SPSS*.

[13] Shivakumar K. M., Prasad S., Chandu G. N. (2009). International Caries Detection and Assessment System: a new paradigm in detection of dental caries. *Journal of Conservative Dentistry*.

[14] World Health Organization (2006). *WHO Child Growth Standards: Training Course on Child Growth Assessment: C. Interpreting Growth Indicators*.

[15] Delgado-Angulo E. K., Hobdell M. H., Bernabe E. (2013). Childhood stunting and caries increment in permanent teeth: a three and a half year longitudinal study in Peru. *International Journal of Paediatric Dentistry*.

[16] Ribeiro C. C. C., Silva M. C. B., Nunes A. M. M. (2017). Overweight, obese, underweight, and frequency of sugar consumption as risk indicators for early childhood caries in Brazilian preschool children. *International Journal of Paediatric Dentistry*.

[17] Chauhan A., Nagarajappa S., Dasar P. L., Mishra P. (2016). Association of body mass index with dental caries among malnourished tribal children of Indore division. *Medicine and Pharmacy Reports*.

